# Preferences for Web-Based Information Material for Low Back Pain: Qualitative Interview Study on People Consulting a General Practitioner

**DOI:** 10.2196/rehab.8841

**Published:** 2018-04-02

**Authors:** Allan Riis, Ditte Meulengracht Hjelmager, Line Dausel Vinther, Michael Skovdal Rathleff, Jan Hartvigsen, Martin Bach Jensen

**Affiliations:** ^1^ Research Unit for General Practice in Aalborg Department of Clinical Medicine Aalborg University Aalborg Denmark; ^2^ Department of Development and Planning Aalborg University Aalborg Denmark; ^3^ Centre for Muscle and Joint Health Department of Sports Science and Clinical Biomechanics University of Southern Denmark Odense Denmark; ^4^ Nordic Institute of Chiropractic and Clinical Biomechanics Odense Denmark

**Keywords:** patient education as topic, medical informatics app, patient participation, general practice, low back pain

## Abstract

**Background:**

Information on self-management, including addressing people’s fears and concerns, are core aspects of managing patients with low back pain (LBP). Web apps with patient information may be used to extend patient-physician consultations and encourage self-management outside of the consultation room. It is, however, important to identify the end users’ needs and preferences in order to maximize acceptance.

**Objective:**

The aim of this study was to identify preferences for the content, design, and functionality of a Web app with evidence-based information and advice for people with LBP in Denmark.

**Methods:**

This is a phenomenological qualitative study. Adults who had consulted their general practitioner because of LBP within the past 14 days were included. Each participated in a semistructured interview, which was audiotaped and transcribed for text condensation. Interviews were conducted at the participant’s home by 2 interviewers. Participants also completed a questionnaire that requested information on age, gender, internet usage, interest in searching new knowledge, LBP-related function, and pain.

**Results:**

Fifteen 45-min interviews were conducted. Participants had a median age of 40 years (range 22-68 years) and reported a median disability of 7 points (range 0-18) using the 23-item Roland Morris Disability Questionnaire. Participants reported that Web-based information should be easy to find and read, easily overviewed, and not be overloaded with information. Subjects found existing Web-based information confusing, often difficult to comprehend, and not relevant for them, and they questioned the motives driving most hosting companies or organizations. The Patient Handbook, a Danish government-funded website that provides information to Danes about health, was mentioned as a trustworthy and preferred site when searching for information and advice regarding LBP.

**Conclusions:**

This study identified important issues to consider when developing and supplementing existing general practice treatment with Web-based information and advice for patients with LBP. Development of a Web app should consider patient input, and developers should carefully address the following domains: readability, customization, design, credibility, and usability.

## Introduction

### Background

With a point prevalence of 9.4% globally, low back pain (LBP) is the health condition that causes the most years lived with disability [1]. All innervated structures in the spine are potential sources of nociception; however, the etiology underlying LBP is often unknown but may include biological, psychological, and social factors [2-4]. Although most episodes of LBP are relatively short, 45% of patients may experience recurrent or persistent pain that causes some to withdraw from work and leisure activities [5,6]. Consequently, people with LBP often consult their general practitioner (GP) for advice. Patient information about staying active, supporting self-management, and removing fears and concerns about LBP are core aspects of evidence-based management [7]. Web apps containing relevant patient information may be used to extend the patient consultation and encourage self-management outside the GPs’ consultation rooms. To ensure the patients’ acceptance and thus, the use of such Web apps, it is important to identify their needs and preferences for the technology.

A recent systematic review highlighted that patient education had positive long-term effects for patients with LBP [8]. Maintaining physical activity and avoiding bed rest can reduce pain and maintain and restore function in acute LBP, whereas behavioral advice can prevent LBP from becoming chronic [9,10]. However, because patients with LBP represent a heterogeneous group, some will, even when receiving evidence-based treatment and advice, have persistent pain [11]; for these patients, information on how to cope with pain is particularly important.

### Web Apps

Information technology–mediated personalized Web apps can improve accessibility and exchangeability of information [12,13]. A personalized approach may address the individual biologic, physiologic, and social factors that are particularly important for the individual patient by addressing the different needs among patients and supporting self-care, which may have long-term effects [14,15]. As such, a Web app tailored to the patient’s profile can differentiate between several types of content (text, pictures, films, and print options) and Web designs.

To inform the developers of Web apps regarding the patients’ preferences, it is essential to involve the end users during the development process. Elucidating barriers and enablers are likely deciding factors for future acceptance and use. Otherwise, patients may find content and design irrelevant and consequently be dissatisfied [16].

### Objective

The aim of this study was to identify preferences for the content and design of a Web app with information and advice for people with LBP consulting a GP.

## Methods

### Design

This was a phenomenological qualitative study based on a constructivist research paradigm. The interview guide was based on methods for designing semistructured interviews [17]. The interview guide was pilot-tested 3 times, resulting in small adjustments (Multimedia Appendix 1).

Additionally, visible, tangible artifacts, as post-it notes and photos, were presented to participants, since these could help to foster a creative environment and support dialog during interviewing [18]. This was performed by giving artifacts to the participants and asking them to be creative during interviewing. The activities were to give insights into the patients’ needs and let them express the knowledge that might be tacit [18]. The visible tangible artifacts were used for 3 activities. In the first activity, post-it notes and a ball pen were handed out to the participants. The purpose of this activity was to gain insight into what knowledge participants found most important in relation to their LBP and use of a Web app. Patients were asked to write one aspect on each post-it note. Following this, patients were handed 6 stickers and asked to prioritize the importance of the post-it notes.

**Figure 1:**
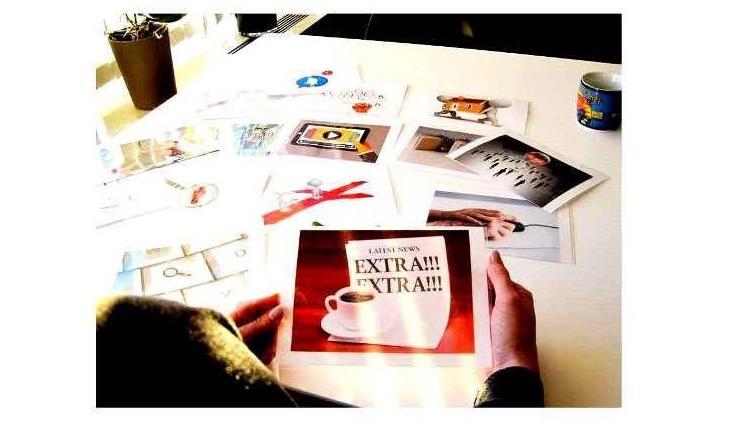
Photos and graphics. Illustration of different photos applied to foster a creative environment and support dialogue. This picture was taken during interviewing at a participants’ home.

The second activity also included post-it notes. The participants’ inputs were discussed to create an overview of objects and techniques used to cope with pain. In some interviews, participants noted down on post-it notes by themselves, whereas in other cases, the interviewers assisted them. In the third activity, laminated cards with photos and graphics (Figure 1) were used as “interview stimuli” [19]. Participants were asked to use these photos as inspiration when describing which aspects they found most important when using Web apps to acquire information related to LBP. Consequently, the artifacts can facilitate participants to select the reference points in the conversation and thus to take lead in inquiry [18]. The photos were placed on the table (Figure 1). Participants were asked to select 3 photos, which they found useful, to support their presentation of important aspects. Besides being an explanation object, the photos contributed to a conversation about positive and negative aspects when using a Web app to obtain information. Furthermore, the variety of photos was selected by the interviewers (LDV and DMH) to support participants in being creative.

The participants were interviewed in their homes by 2 interviewers. The interviews were audiotaped and transcribed without fill-words, but pointing to pictures or objects, and any recorded sounds that influenced the conversation were noted. Interview data were analyzed inspired by a thematic approach in 6 phases [20].

**Figure 2:**
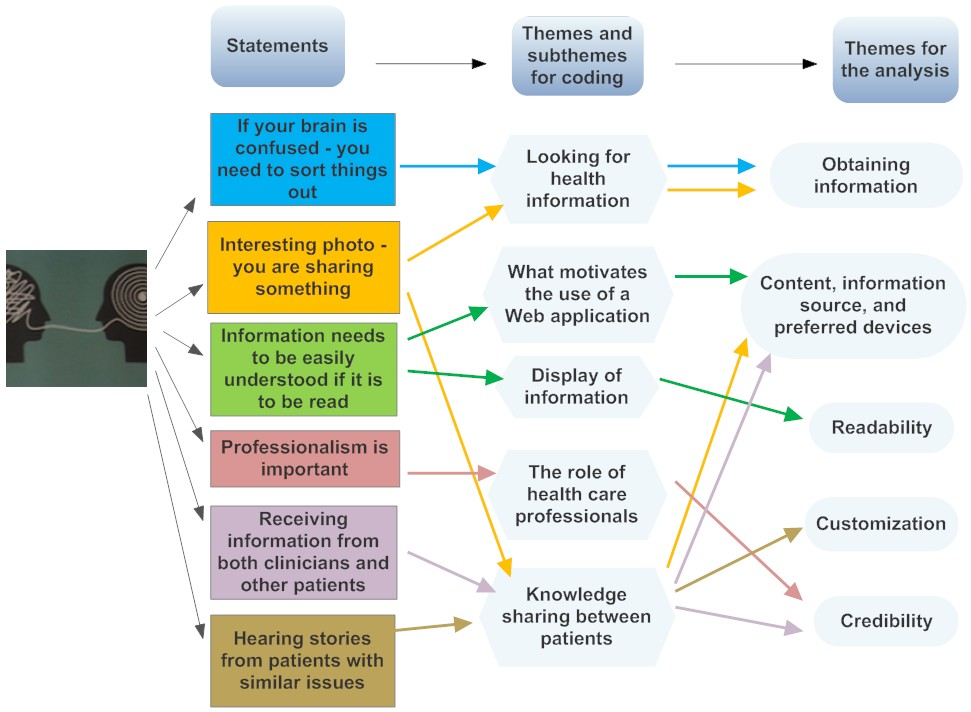
Coding of statements in relation to the photos. The process starting from commenting on a photo, to coding the comment in NVivo, and finally including the comment in the themes for the analysis. The processes are illustrated by unique colors for each statement.

In phase one, data from audio recordings were transcribed by LDV and DMH. Followed by reading the transcriptions and writing down 2 sets of initial ideas for coding, LDV and DMH wrote their combined ideas for coding, and AR noted his suggestions for coding. In phase two, the 2 initial ideas for coding were discussed and consensus for coding themes was agreed upon (Multimedia Appendix 2). In phase three, annotations were gathered under the coding themes, including inputs from the chosen photos during interviews (by AR, LDV, and DMH), allowing annotations to occur under multiple coding themes (Figure 2). In phase four, the themes were reviewed by AR and LDV (or AR and DMH) checking whether the themes worked in relation to the coded extract and with special attention to not missing important information during coding. In phase five, the themes were refined, with special attention to the aim of this study, to the themes being presented in the analysis (Multimedia Appendix 2). In phase six, the final adjustments were made to the analysis by all authors with special focus on translations of quotations from Danish to English.

The study population was balanced between the 3 Stratified Targeted Treatment (STarT) Back groups, with the purpose of including patients with heterogeneous bio-psycho-social profiles and variation in response to commonly used treatment strategies [21]. Following the interviews, participants were given a combined questionnaire that included baseline information regarding age, gender, risk of poor prognosis, use of the STarT Back Tool (SBT), [22], pain duration, pain intensity (Numerical Pain Rating [23]), responses to the Roland Morris Disability Questionnaire [24], and health-related internet search behavior. Reporting followed the standards for reporting qualitative research [25]. A study protocol for this study has previously been published [26].

### Research Group Characteristics

The 2 interviewers (DMH and LDV) did not have any private or clinical knowledge of the respondents other than knowing that the individuals being interviewed met the inclusion criteria. The researchers involved in this project encompass a broad range of professional backgrounds, including a bachelor of radiography and master of techno-anthropology student (DMH), a bachelor of techno-anthropology and master of techno-anthropology student (LDV), a GP practitioner (MBJ), a chiropractor (JH), and 2 physiotherapists (AR and MSR). The authors had expected that participants would have found it difficult to find Web-based information suited to them. The authors had also expected that Web-based information would be reported to be provided in a boring manner and described as time-consuming to read long passages of text before reaching the essential information. Our research is aimed to support the development of guideline-concordant Web-based information. Consequently, a description of requested content compromising this aim has partly been omitted from reporting.

### Context

This study included people aged older than 18 years who were consulting their GP because of LBP of longer than 14 days’ duration. People without access to the internet were excluded, as were pregnant women, people who did not speak Danish as their native language, or those who had signs of a serious underlying disease.

### Sampling Strategy

A physiotherapist, a GP, or a medical staff member acted as a recruiter and invited potential participants, including people currently consulting general practice, people who had previously consulted general practice regarding LBP (cold list recruitment), or people who had recently been referred from general practice to a physiotherapist. The recruiters recorded the contact information and gave it to AR, who contacted eligible participants to make appointments for interviews. AR provided verbal information by phone, and the participants received written information before being interviewed. AR was responsible for including a heterogeneous group of patients with 5 patients from each STarT Back group to ensure variation in bio-psycho-social profiles. Before interviewing, it had been decided to include more than 15 participants to inform us regarding our secondary purpose, “differences in preferences between SBT groups,” if needed. However, following the 15 interviews, no distinctive patterns between the SBT groups were identified. Thus, increasing the sample size did not seem likely to add knowledge about clear differences and was consequently not performed.

### Approval and Ethics

This study was approved by the Danish Data Protection Agency (registration number 2015-57-0001) and conducted according to the principles of the Declaration of Helsinki. The study was not registered with the local ethics committee, as this was not required for interview studies. Participants gave written informed consent.

### Approach

The interviews occurred in people’s homes because that is where the participants cope with their pain and everyday life, and where the information and technology they use most often is present. The 2 interviewers (DMH and LDV) provided written information and collected written informed consent from the patients. After the interviews, people were asked to complete a questionnaire in the presence of the interviewers, who did not assist in filling in the information [26].

### Data Analysis

Participant characteristics determined from the questionnaires [26] were presented as numbers (%) for categorical variables, and mean values (range) for continuous variables. The interviews were analyzed according to a phenomenological approach and using an interpretative analysis to identify preferences for the content and design of a Web app. Furthermore, the study population was divided into 3 groups according to the STarT Back risk groups, and differences between the groups were explored. The coding of the interviews was performed using the NVivo software package (QSR International Pty Ltd, Victoria, Australia).

## Results

### Participant Characteristics

Between October 4, 2016 and January 11, 2017, 15 interviews were conducted. The study population consisted patients with heterogeneity in their baseline characteristics (Table 1).

The interviews lasted approximately 45 min. The initial reading of the transcriptions yielded 16 potential coding themes that during the recoding for the final themes, and subsequently coding for the analysis, were reduced to 7 themes for the analysis (Multimedia Appendix 2).

### Obtaining Information

This theme consisted of earlier experiences and expressed preferences for obtaining information regarding LBP.

Some participants trusted the GPs to supply the necessary information, which was their explanation for not searching for information themselves:

When visiting the GP, you trust him to provide you with relevant information.

Interview 2

Another participant expressed:

The insight [into LBP] is just as important to me and not only to the GP’s...When you understand, it’s possible to take action yourself.

Interview 3

Participants agreed that finding a health information technology (HIT) that encouraged self-management would provide inspiration about what they can do themselves. In general, participants showed skepticism regarding using existing HIT apps; as one participant explained:

You can easily end up looking like a hypochondriac. I think it would seem like that to me.

Interview 1

Existing HITs were mainly associated with information explaining symptoms and diagnostics, which participants found hard to navigate through. Participants did not consider their professional judgments sufficient to relate to the Web-based information. They felt it only made them more confused and frustrated. However, participants were aware of existing HIT apps. The most frequently mentioned were the information site “Net-doktor.dk” (privately owned and financed by advertisements) and “the Patient’s Handbook” at the “Sundhed-dk” (the National Danish eHealth portal financed by the Danish government, which provides access to information for the public and for health care professionals). Some participants associated “Netdoktor.dk” with “serious” conditions such as heart diseases rather than with LBP. Some participants were advised by their GP to look up “Patient’s Handbook” but found the webpage hard to use:

It was difficult for me to find the specific exercises. I had to be persistent – I think others might have given up.

Interview 14

The Patient’s Handbook was, however, the most frequently visited site. HIT apps related to health care professions, such as chiropractors and physiotherapists, were mentioned as relevant, although not often visited. Facebook had introduced some participants to information on both exercises and health care professionals. However, information on Facebook was described as having a low degree of credibility; patients used Facebook as inspiration when the information might result in less pain:

Not everything on the internet is rubbish; even though it seemed unreliable, I thought I might as well try it.

Interview 8

**Table 1:** Baseline characteristics.

Baseline characteristics^a^ of patients with low back pain		Value
Number of participants, N		15
Age in years, median (range)		40 (22-68)
**Gender, n (%)**		
	Male	11 (73)
STarT^b^ Back Tool risk-group		5 in each group
Pain duration > 12 weeks, n (%)		9 (60)
Pain score^c^, median (range)		4 (1-8)
Functional Disability score^d^, median (range)		7 (0-18)
Health information seeking behavior on the internet, monthly or more; n (%)		5 (33)

^a^N=15, questionnaires were filled-in during interviewing. However, for 3 participants, the SBT was reported over the phone to balance participants between the 3 SBT risk-groups (low, medium, or high).

^b^STarT: Stratified Targeted Treatment.

^c^Numerical Pain Rating (0-10, 0=no pain).

^d^Roland Morris Disability Questionnaire (23 items, Patrick version).

### Content, Information Source, and Preferred Devices

This theme consisted of earlier experiences with seeking health information on the internet on different devices.

Participants would rather ask GPs than use the internet for information with regard to a diagnosis:

I would rather talk to a professional when it concerns a clarification [of the cause] who I can sit in front of and ask questions if necessary.

Interview 1

However, regarding whether participants used an HIT app to find information, it would make a difference if GPs recommended it. Furthermore, if GPs recommended an HIT app, participants felt it would save them time searching online. One participant used the term “jungle” about the internet, meaning it is time-consuming to find what is requested because the World Wide Web contains tons of information that may be irrelevant:

Maybe it would increase the interest in the particular webpage, since there are hundreds [of webpages] to choose from. Knowing which one to use, you do not have to go through all to figure out which one is the best.

Interview 4

Participants used a range of devices such as personal computers, smartphones, and tablets when searching online. They argued that their smartphone was always within reach, whereas a computer or tablet was useful when reading larger pieces of text.

### Readability

This theme comprised earlier experiences with reading and understanding Web-based information, including preference to support this in Web apps.

Participants found language style important on HIT apps. One participant expressed:

When a health care professional explains, I don’t understand all of it. Not to be rude – but not all healthcare professionals are able to present information which can be understood, and then it ends up being gibberish [to me], and I will exit the homepage.

Interview 1

A participant described that language should be:

...understandable, like the language non-professionals use: Even though I work in healthcare and am familiar with some Latin, I’m challenged when encountering a lot of [difficult words].

Interview 5

Participants suggested a textbox explaining the Latin words, as this could ease the reading, and having professional text writers do the writing. Participants also stated the importance of considering colors on the webpage because certain color combinations reduce readability.

### Customization

This theme consisted of earlier experiences with the ability of Web apps to meet their needs and suggestions to include this in a Web app for patients with LBP.

A combination of text, photos, and videos was preferred. However, the content sets the bar as to how the information should be presented:

Sometimes when information is presented in text, people do not understand it the way it is intended. In these cases, videos and photos are useful.

Interview 3

Additionally:

some exercises cannot be explained [with text]; they need to be demonstrated.

Interview 13

Participants found text useful when it reinforced explanations in videos. It was likewise described as beneficial to have the opportunity to print out pictures of exercises. Videos were requested as a means to show and explain exercises, as one participant explained:

It would be nice to have someone who knows what he or she is talking about to show an exercise.

Interview 4

Like photos, videos were also argued as a relevant method to make the presentation of information more interesting, especially for people who prefer to learn via visual impressions. It was suggested to let someone explain certain topics, ie, explaining while drawing on a whiteboard. Notifications on when to do exercises were suggested as a part of the HIT app:

I have actually considered setting an alarm on my phone to remind me to do my exercises.

Interview 3

One participant said:

Send an email with the link. This would also serve as a reminder to me.

Interview 14

### Design

This theme comprised earlier experiences with the design of Web apps and suggestions to the design of a Web app for patients with LBP.

Aspects such as first impressions, customization, and certification were considered important to the design and appearance of an HIT app. The purpose of a webpage should be easy to detect at first glance, since participants described a webpage where the purpose is unclear as messy. Participants expressed how a messy front page could make them leave without further interaction with the content. Participants found it critical if Web apps were not suited for the target group:

They have just made a web app and presented the information they believe is relevant.

Interview 8

Another participant suggested a front page prescribing the content and asking questions to guide the information delivered through the Web app. However, it was emphasized that if questions are asked, the reason to ask questions must be clear:

I am the one searching for information; why do they need information?

Interview 8

It was suggested that all text should be presented on one page to avoid clicking around and ultimately getting lost. Others suggested a table of contents as seen in a book, which could provide an easy overview of the HIT app. One participant said:

The application should be user friendly – not too much confusion and use of different colors – it needs to be easy and simple. You do not want to spend too much time looking for the information relevant to you.

Interview 4

### Credibility

This theme comprised earlier experiences with the credibility of Web apps and suggestions to support the trustworthiness of a Web app for patients with LBP.

Participants expressed that layout and text should clearly indicate professionalism. One participant commented on presenting health care professionals as the source of information:

It could work as a certification mark, indicating that someone capable has been a part of it, thereby informing the user whether it is The Health Authority or somebody else certifying the application. It would be something I would look for if it existed.

Interview 8

Participants agreed that other health care professions such as nurses and physiotherapists were reliable sources on equal terms as GPs when information should be presented on the Web. Participants found advertisements on an HIT app bad, as they could send mixed signals and be disturbing:

If advertisements are not related to LBP, it would be particularly strange to present them on the web page.

Interview 4

Additionally:

If the page is stuffed with advertisements, then someone else has an interest in the page – one related to possible profit.

Interview 6

It was argued that if a “wonder cure” had actually been found, why are people not receiving it from their GP already? However, one participant indicated that advertisements could be acceptable if they excluded people from paying for access. In addition to pop-ups, he did not mind advertisements:

Rather advertisements than having to pay for the content.

Interview 8

### Usability

This theme consisted of earlier experiences with the usability of Web apps, in particular the importance of avoiding the sense of getting lost.

Participants stated that a search function is desirable to have on an HIT app. However, one participant expressed when using search functions in general:

It is not something I do very often, though I know the possibility is there, simply because I don’t want to spend my time on it, as I think I find a lot of information with no relevance to me.

Interview 9

Additionally:

A good search function is one which understands what I mean, because I don’t know all the Latin expressions to my back problems.

Interview 3

Participants explained how a search function becomes crucial if the HIT app is hard to navigate. An alternative to a search was some kind of guide to what the HIT app contains.

## Discussion

### Principal Findings

Participants considered a Web app potentially useful in combination with advice and information regarding LBP provided by their GP. However, certain barriers prevent most patients from frequently using the internet as a source of health care information. The domains of readability, customization, design, credibility, and usability are all important for patient satisfaction with a Web app.

### Comparison With Prior Work

The credibility of the provider was found to be a key determinant for considering Web-based health information to be trustworthy, which was also identified by Eysenbach et al [27]. Most of the requested information or content, such as seeking a diagnosis (also if the diagnosis is nonspecific LBP), information about possible prognosis, advice about how to stay active, and advice on how to perform exercises, is in accordance with international guidelines for what is recommended to be delivered by health care professionals [28,29]. However, in this study, people with LBP also preferred receiving more individualized information, especially on how to cope with pain, and how to choose and perform exercises. In a Web app, this could be achieved by integrating advice on pain and exercises according to the principle described by Silbernagel et al [30]. They proposed a continuous pain monitoring model to motivate and guide the rehabilitation of patients with Achilles tendinopathy [30]. Combining guideline-concordant advice with the tailoring of content to fit users’ preferences and interests can be an effective tool in self-management of LBP [28]. This has previously been found to be a useful tool to achieve the initial use of the technology; however, as also previously reported, it is unclear whether this leads to satisfied users and continuous user engagement [31].

### Clinical Implications

In this study, patients expressed the need for Web-based information for LBP. Some patients had problems with understanding the content, whereas patients understanding the content found the content on existing Web apps irrelevant to them. Therefore, an effort to involve patients in the development of Web apps, include patients’ preferences, and thereby increasing satisfaction with Web apps for LBP, have a large potential to increase the use of Web apps. An increased use can lead to improved functional outcomes for patients with LBP, the condition most prevalent among all health care conditions worldwide [1].

### Strengths and Limitations

The 15 patients in this study were interviewed when they had an appointment to see their GP; therefore, they were at a point in time when they actively sought information regarding LBP. Furthermore, interviewing took place at the patients’ homes, thereby reflecting the settings, where patients normally seek information on the Web. Timings combined with settings are unique and strengthened this study. Patients were sampled with the purpose to reach maximum variation in bio-psycho-social profiles by use of the SBT [22], which strengthens the generalizability of findings to other primary health care settings.

Only one-third of the responders sought information monthly or on a more frequent basis, which may have restricted the findings regarding Web app usage and thereby weakened parts of the analysis. It had been planned to describe differences between the 3 SBT groups; however, based on this material, it was not possible to draw any conclusions. A larger sample size may be needed to identify differences. This study was performed to inform the development of informational material to supplement routine care. Participants were informed of this before the interviews. Consequently, this strategy does not support transferability to settings in which the purpose of the information is a stand-alone intervention.

### Conclusions

This study identified important issues to consider when developing and supplementing existing general practice treatment with Web-based information and advice to patients with LBP. Important domains to address in the development of a Web app for people with LBP are readability, customization, design, credibility, and usability. Some of the findings were consistent with our expectations. However, the inability among eHealth providers to inform in language suited to the patients surprised us. The authors were also surprised that patients often felt that the available information did not relate to their condition.

## Appendix

Multimedia Appendix 1 Interview guide.

Multimedia Appendix 2 Themes for coding and analysis.

## Abbreviations

GP: general practitioner
HIT: health information technology
LBP: low back pain
STarT: Stratified Targeted Treatment
SBT: STarT Back Tool
