# Supplementary material for: Preferences for Web-Based Information Material for Low Back Pain: Qualitative Interview Study on People Consulting a General Practitioner
Source: JMIR Rehabil Assist Technol. 2018 Apr 2;5(1):e7. doi: 10.2196/rehab.8841 (PMC5902697; doi:10.2196/rehab.8841)
Supplement: Multimedia Appendix 1 [file rehab_v5i1e7_app1.pdf]

Research question	Possible interview questions
**Presentation**	Who are we?<br><br>Who are you? (What is low back pain to you, children, education, etc.)
**Importance of low back pain**	Mention what is important to you. Write issues down. Prioritise among the issues<br><br>Why are these (3 highly prioritised issues) important to you?
**Influence of low back pain**	How does low back pain influence you and your activities?
**Self-management of low back pain**	(Referring to the written issues)<br><br>Tell or show us what you do when you have pain<br><br>Why do you do this?<br><br>Where did you get this knowledge/idea?
**Understanding of low back pain**	Do you know the reason for your low back pain?<br><br>Do you look for explanations?<br><br>Do you look for information?<br><br>Which kind of information do you seek?
**Needed**	Do you search for information on the Internet?

**information**	Which kind of information do you search for?<br><br>Do you search for what you can do yourself?<br><br>How should information be if it is suited for you?<br><br>How important is it that information on the Internet is aimed at you?
**Source of information**	Where do you search for information (Internet, books, etc.)?<br><br>What is good and bad about these sources?<br><br>Which technologies do you use (PC, tablet, smartphone, etc.)?
**A web application**	When you enter a homepage, what is most important to you?<br><br>Explain to the patient about this project:<br>1. We aim to develop a homepage for low back pain<br>2. The web application is aimed at patients consulting their GP for advice on low back pain<br>3. The web application is an add-on to existing treatment options for low back pain in primary care<br>4. It is important that the web application is suited to you<br><br>(Showing the patient different illustrations)<br><br>Part 1: Among these illustrations, pick three that can help to explain what is important for a web application to you.

	Part 2: Can you find other illustrations that can explain why you will stay on a homepage or illustrations explaining why you will exit the homepage?<br><br>How should information be presented to you?
**Credibility of a homepage**	When do you ask a healthcare professional for advice?<br><br>When do you seek information by yourself?<br><br>What is the difference between the two?
**Network**	Can other patients with low back pain be helpful to you?<br><br>How can networks help you?
**What is important for a patient with low back pain**	If one of your friends or colleagues experienced low back pain for the first time and you were to give him/her a present, what kind of present should this be?
**Conclusion**	Completion of the questionnaire (excl. STarT)<br><br>Can we contact you if we have any further questions?<br><br>Thanks for your participation!
