# Supplementary material for: Preferences for Web-Based Information Material for Low Back Pain: Qualitative Interview Study on People Consulting a General Practitioner
Source: JMIR Rehabil Assist Technol. 2018 Apr 2;5(1):e7. doi: 10.2196/rehab.8841 (PMC5902697; doi:10.2196/rehab.8841)
Supplement: Multimedia Appendix 2 [file rehab_v5i1e7_app2.pdf]

Initial potential themes for coding	Themes and sub themes for coding	Themes for the analysis
**Patients' health problems**	**Looking for health information**	Obtaining information
**Patients knowledge**	- **In which degree?**	Content, information source, and preferred devices
**Requested knowledge**	- **How?**	Readability
**Current access to low back pain (LBP) information**	- **Special needs?**	Customisation
**How do patients change behavior?**	**Deciding factors for using health info online**	Design
**Would cause change in health behavior?**	- **Barriers**	Credibility
**Are they currently looking for information online?**	- **Facilitators**	Usability
**Applicability of current information on LBP**	**Knowledge sharing between patients**	
**Current use of different devices**	**The role of healthcare professionals**	
**Important features to support the use of online info**	**Display of information**	
**Which elements is requested for a web application**	**What motivates the use of a web application / homepage**	
**The role of a web application vs the role of healthcare professionals**	**Technological requirements to a web application**	
**Networking between patients**	- **Negative aspects**	
**Do patients thrust information**	- **Positive aspects**	
**Interactivity and motivation**	**Design of a web application**	
**Which sites are currently being accessed**	**Knowledge about LBP**	
	- **Expressed needs for information about LBP**	
	- **Existing knowledge**	
